# Diminishing Endograft Apposition during Follow-Up Is an Important Indicator of Late Type 1a Endoleak after Endovascular Aneurysm Repair

**DOI:** 10.3390/jcm12123969

**Published:** 2023-06-10

**Authors:** Roy Zuidema, Anna C. M. Geraedts, Willemina A. van Veldhuizen, Sana Mulay, Jean-Paul P. M. de Vries, Richte C. L. Schuurmann, Ron Balm, the ODYSSEUS-T1EL Study Group

**Affiliations:** 1 Department of Surgery, Division of Vascular Surgery, University Medical Center Groningen, 9713 GZ Groningen, The Netherlands; w.a.van.veldhuizen@umcg.nl (W.A.v.V.); j.p.p.m.de.vries@umcg.nl (J.-P.P.M.d.V.); r.c.l.schuurmann@umcg.nl (R.C.L.S.); 2 Department of Surgery, Amsterdam University Medical Centers, University of Amsterdam, Amsterdam Cardiovascular Sciences, 1081 HV Amsterdam, The Netherlands; a.c.geraedts@amsterdamumc.nl (A.C.M.G.); s.mulay@amsterdamumc.nl (S.M.); r.balm@amsterdamumc.nl (R.B.)

**Keywords:** aortic aneurysm, abdominal, endovascular procedures, endoleak

## Abstract

Late type 1a endoleaks (T1aELs) after endovascular aneurysm repair (EVAR) are hazardous complications which should be avoided. This study investigated the evolution of the shortest apposition length (SAL) post-EVAR and hypothesised that a declining apposition during follow-up may be an indicator of T1aEL development. Patients with a late T1aEL were selected from a consecutive multicentre database. For each T1aEL patient, the preoperative computed tomography angiography (CTA), first postoperative CTA, and pre-endoleak CTA were analysed. T1aEL patients were matched 1:1 to uncomplicated controls, based on endograft type and follow-up duration. Anatomical characteristics and endograft dimensions, including the post-EVAR SAL, were measured. Included were 28 patients with a late T1aEL and 28 matched controls. The SAL decreased from 11.2 mm (5.6–20.6 mm) to 3.9 mm (0.0–11.4 mm) in the T1aEL group (*p* = 0.006), whereas an increase in SAL was seen in the control group from 21.3 mm (14.1–25.8 mm) to 25.4 mm (19.0–36.2 mm; *p* = 0.015). On the pre-endoleak CTA, 18 patients (64%) in the T1aEL group had a SAL < 10 mm, and one (4%) patient in the control group had a SAL < 10 mm on the matched CTAs. Moreover, three mechanisms of decreasing sealing zone were identified, which might be used to determine optimal imaging or reintervention strategies. Diminishing SAL < 10 mm is an indicator for T1aEL during follow-up, it is imperative to include apposition analysis during follow-up.

## 1. Introduction

Endovascular aneurysm repair (EVAR) has become an important treatment option for patients with an abdominal aortic aneurysm (AAA). With the development of new generation endografts, the safety and durability of the procedure has greatly improved [1,2]. Despite these developments, type 1a endoleaks (T1aELs) are still the Achilles’ heel of modern EVAR [3]. A T1aEL is a leak at the proximal attachment site of the endograft, due to an insufficient sealing zone, which results in persistent blood flow into the aneurysmal sac. Late T1aELs, which occur at least >90 days post-EVAR, are particularly hazardous, because they are difficult to foresee and can result in unexpected aneurysm rupture [4].

To detect these postoperative endoleaks and endograft migration, the guidelines of the Society for Vascular Surgery and the European Society for Vascular Surgery recommend various follow-up regimens, including regular computed tomography angiography (CTA) scans [5,6]. Currently, these protocols are mostly aimed at detecting postoperative complications. However, this approach does not use the full potential of these CTA scans. Instead of detecting complications, it would be better if complications could be foreseen and prevented. Andersson et al. recently published a study in which they stated that precursors of aneurysm rupture are missed during routine follow-up, due to the lack of a structured CTA analysis protocol [7]. Meticulous and consistent analyses of the real achieved sealing zone, the length from the proximal endograft fabric to where the endograft is no longer circumferentially apposed to the aortic wall, might aid in the detection of these precursors [7,8].

Vascular imaging analyses (VIA) software has been developed to detect small changes in endograft apposition on CTA scans post-EVAR [9]. By using VIA, it is possible to quantify the post-EVAR apposition and its evolution over time [8]. As a result, it is possible to detect upcoming T1aELs before they are visible on a CTA-scan [10]. The current study aimed to confirm this statement in a consecutive cohort with long-term follow-up, and hypothesised that a decline of apposition during follow-up precedes the development of a late T1aEL. The secondary objectives were to quantify postoperative aortic neck enlargement and to determine the causes of decreasing sealing zone.

## 2. Materials and Methods

### 2.1. Study Design

This retrospective multicentre case-control study used data of patients from the ODYSSEUS study [11]. The ODYSSEUS study was a national multicentre retrospective cohort study and was granted approval by the Amsterdam University Medical Centres Medical Ethics Review Committee. This study was conducted in compliance with the STROBE guidelines [12].

### 2.2. Patients

Inclusion criteria were based on a previous study by this research group regarding apposition on the first postoperative CTA, with largely the same patients [13]. The late T1aEL group included patients with a first post-EVAR CTA (<90 days post-EVAR) without a type 1 (or type 3) endoleak, who developed a T1aEL thereafter. Patients with complex EVAR procedures (e.g., fenestrated or branched repair) or proximal adjuncts (e.g., EndoAnchors (Medtronic)) were excluded. Patients had to have at least one follow-up CTA in addition to the first postoperative CTA. Compared with our previous study, six patients were excluded due to the lack of multiple follow-up CTAs, and two additional patients could be included because the absence of a preoperative CTA was not an exclusion criterion for the current study. Supplementary Figure S1 provides an overview of the selection process of the T1aEL patients.

For each included T1aEL patient, the preoperative CTA, first post-EVAR CTA, pre-endoleak (last uncomplicated) CTA, and the CTA with the endoleak were retrieved. Subsequently, T1aEL patients were matched 1:1 with controls without T1aEL, based on the endograft type and follow-up duration between the EVAR procedure and the pre-endoleak CTA. These patients were selected from the remaining ODYSSEUS cohort. It was difficult to effectuate comparable follow-up, so the patients who were treated with an Endurant (Medtronic Cardiovascular, Santa Rosa, CA, USA) endograft were selected from an uncomplicated Endurant cohort [14].

### 2.3. Measurement Protocol and Endpoints

All CTAs were analysed in 3mensio 10.1 software (Pie Medical Imaging BV, Maastricht, The Netherlands). The entire CTA measurement protocol has been published [13]. On each preoperative CTA, the neck diameter, intended oversizing, neck length, infrarenal/suprarenal angulation, neck thrombus, neck calcification, neck shape, and maximum aneurysm diameter were measured. The neck diameter was defined as the diameter from adventitia to adventitia at the level of the lowest renal artery. In addition, diameters on multiple levels relative to the lowest renal artery were measured. Each individual diameter was calculated as the average of two perpendicular diameters. Intended oversizing was calculated as (nominal endograft diameter/pre-EVAR neck diameter − 1) × 100% [14]. Neck length was defined as the length over the centreline, starting from the level of the lowest renal artery to the level where the aortic diameter was increased 10%, compared with the neck diameter. Infrarenal and suprarenal angulation were measured over the centreline using the centreline angle tool. The presence of neck thrombus and calcification (>25% circumference of the aortic neck) were assessed at baseline. Neck shape was classified as hostile for conical, barrel, or dumbbell shaped necks.

All postoperative endograft dimensions were calculated in VIA software (Endovascular Diagnostics BV, Utrecht, The Netherlands) using the centreline, aortic mesh, and three-dimensional coordinates, according to previously published and validated methods [9,10,13]. The primary endpoint, the real achieved sealing zone, was defined as the length starting at the proximal end of the endograft fabric and over which the endograft material has proper circumferential apposition with the aortic wall [8]. The shortest apposition length (SAL) was used to quantify the real achieved sealing zone. SAL was defined as the shortest length between the proximal end of the endograft fabric to the first level where the endograft lost circumferential apposition with the aortic wall [9]. Patients will be classified as high risk when the SAL is <10 mm, based on the existing literature and device instructions for use [13,15,16]. The SAL/aortic neck length ratio, shortest fabric distance, and endograft expansion were also calculated. The SAL/aortic neck length ratio was used to determine which part of the preoperative neck was actually sealed [14]. The shortest fabric distance is the shortest length between the proximal end of the endograft fabric to the lowest renal artery. Expansion was calculated as the expanded endograft diameter/original main body diameter × 100% [9]. As a secondary end point, diameters at eight aortic levels relative to the lowest renal artery baseline were measured as well (+40 mm, +30 mm, +20 mm, +10 mm, at baseline, −10 mm, −15 mm, and −20 mm).

### 2.4. Statistical Methods

Data were collected in REDCap (Vanderbilt University, Nashville, TN, USA) and analysed in IBM SPSS Statistics 23.0 software (IBM Corporation, Armonk, NY, USA). All data were determined as not normally distributed through visually inspected histograms and quantile–quantile plots. Therefore, all variables are expressed as median with interquartile range. Data were compared between the groups and between follow-up assessments within one group. Differences in categorical data were tested using the Chi-square test. Differences in continuous unpaired data were tested using the Mann–Whitney *U* test and paired data with the Wilcoxon signed rank test. A *p* value was considered statistically significant when the two-tailed α was ≤0.05.

## 3. Results

### 3.1. Baseline and Follow-Up Characteristics

The study included 56 patients, 28 patients with a T1aEL, and 28 uncomplicated matched controls. All patients underwent EVAR between 2007 and 2016, the median age was 70 years (65–75 years), and 48 patients were male (86%). In each group, 18 patients were treated with an Endurant (Medtronic) endograft (64%), 4 (14%) with Zenith (Cook Medical, Bloomington, IN, USA), 3 (11%) with Talent (Medtronic), and 3 (11%) with Excluder (W. L. Gore & Associates, Flagstaff, AZ, USA) endografts. The first postoperative CTA was made 28.0 days (13.0–40.8 days) post-EVAR in the T1aEL group, compared to 32.5 days (30.0–42.8 days) post-EVAR in the control group (*p* = 0.79). No significant differences were found for the time between EVAR and the pre-endoleak (last uncomplicated) CTA in the T1aEL group (27.5 months (14.0–67.5 months)) versus the time between EVAR and the matched CTAs in the control group (41.5 months (19.0–61.5 months); *p* = 0.42). The time between EVAR and the CTA with the T1aEL was 65.0 months (45.0–84.8 months) compared with 62.0 months (43.0–73.3 months) for the last CTA/DUS follow-up assessment in the control group (*p* = 0.42).

Preoperative anatomical characteristics are presented in Table 1. Neck diameter (26.4 mm (24.3–29.6 mm) vs. 23.1 mm (22.3–24.7 mm); *p* <0.001) and endograft diameter (30.5 mm (28.0–36.0 mm) vs. 28.0 mm (25.0–29.5 mm); *p* = 0.001) were significantly larger in the T1aEL group. The presence of a hostile shape was more frequent in the T1aEL group (22 (84.6%) vs. 14 (50.0%); *p* = 0.007. Eight patients had a preoperative neck length <10 mm in the T1aEL group, which is outside IFU, compared to four patients in the control group. Seven patients had a preoperative neck diameter >28 mm in the T1aEL group, compared to two patients in the control group.

On the completion angiography, four patients in the T1aEL group had a type 2 endoleak, compared to five patients in the control group. Whereas on the first postoperative CTA, eight patients in the T1aEL group had a type 2 endoleak, compared to one patient in the control group.

### 3.2. Postoperative Endograft and Aneurysm Dimensions

Table 2 provides an overview of the absolute post-EVAR endograft and aneurysm dimensions, and Table 3 summarizes the differences of these values between the first postoperative CTA and pre-endoleak/matched CTAs. No significant baseline differences were found between the groups for the shortest fabric distance (*p* = 0.62), graft expansion (*p* = 0.082), and maximum aneurysm diameter (*p* = 0.23) at the first postoperative CTA. The SAL at the first post-EVAR CTA was significantly shorter in the T1aEL group (11.2 mm (5.6–20.6 mm)) compared with the control group (21.3 (14.1–25.8 mm); *p* = 0.002). The SAL/aortic neck length ratio was significantly lower in the T1aEL group compared with the control group (0.6 (0.3–1.1) vs. 0.8 (0.6–1.8); *p* = 0.046).

**Figure 1:**
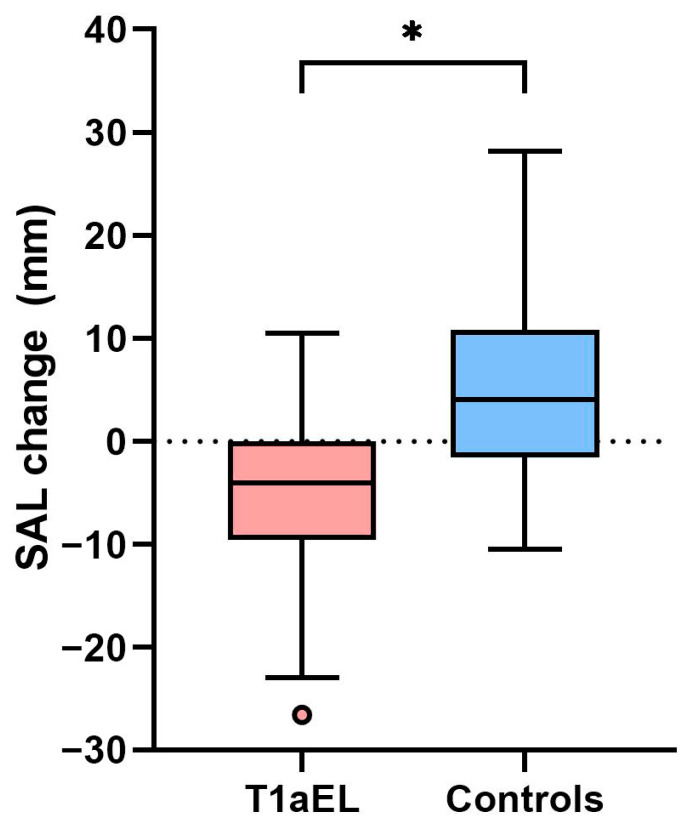
Shortest apposition length (SAL) change between the first postoperative computed tomography angiography (CTA) and the pre-endoleak/matched CTA. Box and whisker plot: the line in the middle of each box indicates the median; the top and bottom borders of the box mark the 75th and 25th percentiles, respectively. The upper and lower whiskers extend from the hinge to the highest value and lowest value, respectively, that is within 1.5 IQR of the hinge, and the circle indicates an outlier. T1aEL: Type 1a endoleak. * *p* < 0.05.

The SAL decreased from 11.2 mm (5.6–20.6 mm) to 3.9 mm (0.0–11.4 mm) in the T1aEL group (*p* = 0.006), whereas an increase in SAL was seen in the control group from 21.3 mm (14.1–25.8 mm) to 25.4 mm (19.0–36.2 mm; *p* = 0.015). Figure 1 shows the SAL change between the first postoperative CTA and the pre-endoleak/matched CTA. The SAL change was significantly different between the groups (−4.0 mm (−9.6 to 0.0 mm) in the T1aEL group vs. 4.0 mm (−1.6 to 10.8 mm) in the control group; *p* = <0.001). The SAL/aortic neck length ratio decreased from 0.6 (0.3–1.1) to 0.1 (0.0–0.4) in the T1aEL group (*p* = 0.001), but did not significantly change in the control group (0.8 (0.6–1.8) vs. 1.0 (0.8–1.9); *p* = 0.076). On the pre-endoleak CTA, 18 patients (64%) in the T1aEL group had a SAL <10 mm, and only one patient (4%) in the control group had a SAL <10 mm on the matched CTA.

The shortest fabric distance significantly increased in both groups. In the T1aEL group, the shortest fabric distance increased from 1.2 mm (−1.3 to 7.4 mm) to 5.7 mm (2.0–12.3 mm; *p* < 0.001), and in the control group from 1.0 mm (0.4–3.5 mm) to 5.0 mm (1.1–9.2 mm; *p* = 0.011). Furthermore, the maximum aneurysm diameter decreased in the control group from 62.8 mm (59.2–66.0 mm) to 52.0 mm (44.0–60.3 mm; *p* < 0.001), but remained unchanged in the T1aEL group (64.3 mm (60.6–73.7 mm) vs. 65.3 mm (57.1–75.6 mm); *p* = 0.77).

### 3.3. Neck Diameters

**Figure 2:**
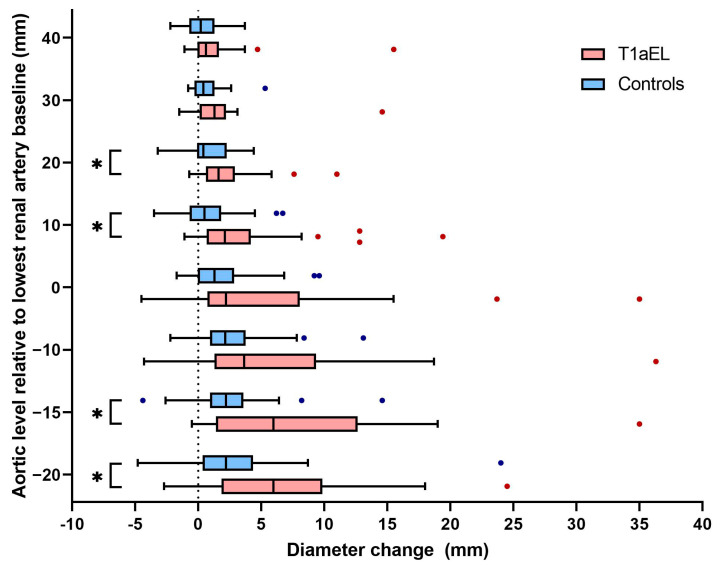
Neck diameter change between the first postoperative computed tomography angiography (CTA) and the pre-endoleak CTA/matched CTA at different aortic levels relative to the lowest renal artery baseline. Box and whisker plot: the line in the middle of each box indicates the median; the top and bottom borders of the box mark the 75th and 25th percentiles, respectively. The upper and lower whiskers extend from the hinge to the highest value and lowest value, respectively, that is within 1.5 IQR of the hinge, and the circles indicate outliers. T1aEL: type 1a endoleak. * *p* < 0.05.

Figure 2 shows the change in aortic neck diameter between the first postoperative CTA and the pre-endoleak/matched CTA at different aortic levels. A significantly larger increase in diameter was seen in the T1aEL group, 20 mm and 10 mm above, and 15 mm and 20 mm below the lowest renal artery compared with the control group (*p* = 0.046; *p* = 0.002; *p* = 0.015; *p* = 0.021, respectively).

### 3.4. Mechanisms of Decreasing Sealing Zone

**Figure 3:**
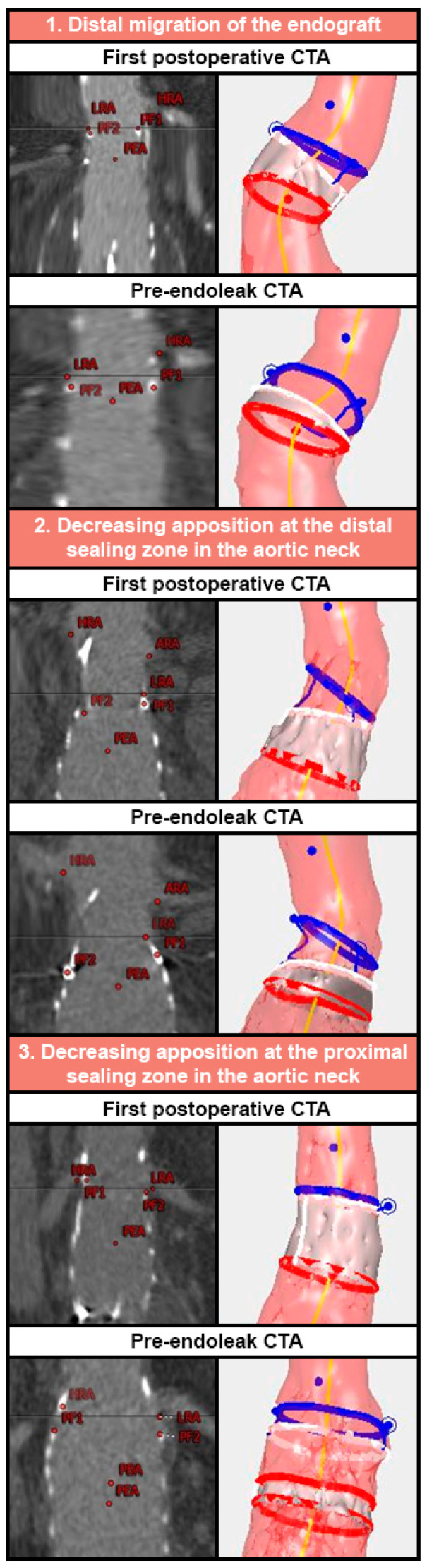
Examples of different mechanisms of decreasing sealing zone on computed tomography angiography (CTA) and the corresponding vascular imaging analyses (VIA) output. The blue circle represents the origin of the renal arteries, the white circle the endograft fabric, and the red circle the first slice where circumferential apposition is lost. Thus, the grey area represents apposition. ARA: accessory renal artery; HRA: highest renal artery; LRA: lowest renal artery; PBA: proximal beginning of apposition; PEA: proximal end of apposition; PF: proximal fabric marker.

By analysing postoperative endograft dimensions, it was possible to identify three different mechanisms of decreasing sealing zone before the T1aEL is actually visible, examples of which are shown in Figure 3. The first mechanism is a relatively unchanged neck with distal migration of the endograft, the second is decreasing apposition at the distal sealing zone in the aortic neck without displacement of the endograft (distal loss), and the third is decreasing apposition at the proximal sealing zone in the aortic neck without displacement of the endograft (proximal loss). On the pre-endoleak CTA of the T1aEL patients we identified six patients with migration, five patients with a proximal loss, four patients with a distal loss, two patients with a proximal loss and migration, and two patients with a distal loss and migration. Five patients had no decrease in apposition and four patients had no apposition at the first postoperative CTA, which made it impossible to classify them.

## 4. Discussion

The results of this study indicate the clinical importance of the determination of the sealing zone post-EVAR. Patients with a late T1aEL demonstrated a diminishing SAL during follow-up, which is not only caused by migration, but can also be caused by decreasing endograft apposition at the distal or proximal sealing zone in the aortic neck. On the contrary, patients with uncomplicated follow-up demonstrated an increasing SAL, most probably due to aneurysm sac shrinkage. It is possible to identify patients at risk for future T1aEls, before the endoleak is actually present, by consistently measuring the SAL during follow-up. Moreover, different mechanisms of a decreasing sealing zone may be detected before the T1aEL is present, which could be used to determine optimal reintervention or follow-up strategies.

Prior studies have noted the importance of measuring the sealing zone on the first postoperative CTA [13,15,16]. They identified SAL < 10 mm as indicator of a high risk for developing T1aEL. A European expert opinion advised considering reintervention in patients with decreasing sealing zone during follow-up, without the presence of visible complications [8]. Unfortunately, clinical evidence is still limited. Schuurman et al. demonstrated, in a different patient cohort, that negative evolution of the sealing zone might be a predictor for T1aEL or migration [10]. Limitations of their study include the fact that the patients had a short median follow-up (<2 years) and that the groups were not matched. The current study confirmed these findings in a different patient group. Even though the preoperative neck length and the SAL at the first postoperative CTA were already relatively short in the T1aEL group, a significantly decreasing SAL was found during follow-up. In addition to the SAL changes, the shortest fabric distance increased in both groups during follow-up, indicating that both groups demonstrated some endograft displacement. However, the maximum aneurysm diameter decreased in the control group while it remained stable in the T1aEL group. Patients in the control group seem to gain an additional sealing zone from the distal part in the aortic neck, due to aneurysm sac shrinkage, whereas future T1aEL patients only lose sealing zone. Almost all uncomplicated patients had a SAL ≥10 mm, whereas a large portion of the T1aEL group had a SAL <10 mm. This study confirms that decreasing SAL could eventually lead to a T1aEL, especially a SAL <10 mm indicates a high risk for T1aEL, either on the first postoperative CTA or during follow-up. It is important to consider that, although not statistically significant, patients in the T1aEL group had a shorter median neck length compared to the control group, which is also reflected in the SAL on the first postoperative CTA. This emphasizes the importance of meticulous preoperative sizing and planning and the risks of performing EVAR in patients with a short neck length. It can be discussed whether patients with a SAL <10 mm should undergo “preventive” interventions to at least stabilize the sealing zone. Naturally, if a reintervention is considered, the risk of developing an actual T1aEL should outweigh the procedural risks of the reintervention.

Whereas early T1aELs are mostly associated with short or severely angulated necks, undersizing, or misplacement of the endograft, determination of the origin of late T1aEL poses a larger challenge [17,18]. Late T1aELs have multifactorial origins, such as distal migration of the endograft, aneurysm growth (due to a primary type 1b, 2, or 3 endoleak), too much oversizing resulting in proximal neck dilatation, or disease progression [3,7,19,20,21,22]. We identified three mechanisms of decreasing sealing zone leading to a T1aEL. The next step would be to determine optimal reintervention strategies in these high-risk patients with a SAL that decreases <10 mm, which could be based on the mechanism of decreasing sealing zone, especially because long-term outcomes of reinterventions for T1aELs are promising [23]. Decreasing apposition at the distal sealing zone in the aortic neck should be distinguished from decreasing apposition at the proximal sealing zone in the aortic neck and endograft migration. Decreasing apposition at the distal sealing zone is mostly due to progressive disease and aneurysm growth. In this case, it is important to look for a primary type 1b, 2, or 3 endoleak. If present, these should be treated first. If no other endoleak is present, it might be worth considering the use of EndoAnchors to secure the remaining sealing zone [24]. However, endograft migration and decreasing apposition at the proximal sealing zone in the aortic neck will require prolongation of the sealing zone by proximal extension of the endograft with an extension cuff or fenestrated device.

A larger increase of the aortic neck diameter on several levels was observed in the T1aEL group compared with the control group. Although the larger increase in the T1aEL group could not be seen on each level, possibly due to the small sample size, a trend toward a larger increase in the T1aEL group was visible for almost all levels. This is an important cause of decreasing apposition in the aortic neck, either a decrease at the proximal sealing zone due to proximal aortic neck dilatation or a decrease at the distal sealing zone due to aneurysm growth and endoleaks. The occurrence of proximal aortic neck dilatation and its increased risk for type 1a endoleak was also reported by Kouvelos et al. [25] and Chatzelas et al. [26]. The postoperative proximal aortic neck dilatation might be due to (too large) oversizing and the radial force of the endograft, or the use of suprarenal fixating endografts [27,28,29]. The current study included a large portion of Endurant and Zenith endografts with suprarenal fixation (75%) in both groups, which might explain the frequent occurrence of proximal neck dilatation. Notably, the neck diameter was significantly larger in the T1aEL group on the preoperative CTA. In our previous study, we demonstrated that a larger preoperative neck diameter is an independent predictor for a late T1aEL [13]. Next to assessment of real achieved sealing zone, aortic neck diameters should be measured during regular CTA imaging follow-up.

### Limitations

Although this study highlighted the added value of measuring apposition during follow-up, several methodological limitations are present. Even though efforts were made to include a large number of T1aEL patients, the sample size was relatively small. Each patient was carefully matched, based on endograft and follow-up duration; however, some form of selection bias was inevitable, and we did not match for all relevant (preoperative) baseline characteristics. To enhance follow-up duration, the control group was supplemented with duplex ultrasound imaging, which might have a lower sensitivity for endoleak detection [30]. For all included patients, the preoperative CTA, the first postoperative CTA, and the pre-endoleak CTA were analysed. As a result of including CTA scans of predefined events, the time between the primary EVAR procedure and the CTA before the endoleak varied between patients, which makes it difficult to attribute a time frame to determined outcomes. This also applies to the time between the pre-endoleak CTA and the CTA with the endoleak, which could vary between several months to >5 years, and might be one of the reasons that not all T1aEL patients demonstrated diminishing SAL on the pre-endoleak CTA. It should also be noted that the preoperative aortic neck diameter (which was significantly larger in the T1aEL group) and the preoperative neck length influence the SAL on the first postoperative CTA. This might be the cause of the significantly shorter SAL on the first postoperative CTA in the T1aEL group. Lastly, since the VIA software is currently not generally available, the most practical method to estimate the postoperative sealing zone at this time is measuring the centreline sealing length.

## 5. Conclusions

This study confirms that diminishing SAL <10 mm is an important indicator for T1aEL during follow-up. As a result, it is possible to identify T1aEL patients before the endoleak is actually present on CTA imaging. In patients with diminishing SAL, especially those with a SAL < 10 mm, a preventive reintervention could be considered, such as EndoAnchors or proximal extension of the sealing zone with an extension cuff or fenestrated device. Alternatively, these patients should at least receive frequent CTA follow-up with apposition analyses. Mechanisms of decreasing sealing zone could be used to determine optimal reintervention or imaging strategies before the T1aEL is present. It is imperative to include assessment of endograft apposition in regular post-EVAR follow-up.

## Figures and Tables

**Table 1:** Preoperative anatomical characteristics.

	Type 1a Endoleak (*n* = 26)	Controls (*n* = 28)	*p*-Value
Neck diameter, mm	26.4 (24.3–29.6)	23.1 (22.3–24.7)	**<0.001**
Endograft diameter, mm	30.5 (28.0–36.0)	28.0 (25.0–29.5)	**0.001**
Intended oversizing, %	11.6 (7.0–25.8)	15.6 (10.6–20.8)	0.52
Neck length, mm	14.0 (9.0–29.9)	25.5 (10.0–34.8)	0.21
Infrarenal angulation, °	46.5 (37.8–61.8)	49.5 (40.3–62.0)	0.66
Suprarenal angulation, °	34.0 (24.8–58.0)	29.0 (16.5–44.5)	0.085
Neck thrombus > 25%	8 (30.7)	9 (32.1)	0.92
Neck calcification > 25%	6 (23.1)	9 (32.1)	0.73
Hostile shape *	22 (84.6)	14 (50.0)	**0.007**
Maximum aneurysm diameter, mm	63.5 (58.9–69.7)	61.4 (58.4–64.0)	0.27
Inside instructions for use	16 (61.5)	21 (75.0)	0.29

Data are presented as median (quartile 1–quartile 3) for continuous data or *n* (%) for categorical data. Boldface *p* values are statistically significant (*p* < 0.05). * Hostile shape is defined as a conical, barrel, or dumbbell shaped neck.

**Table 2:** Absolute post-endovascular aneurysm repair endograft and aneurysm dimensions.

	Type 1a Endoleak (*n* = 28)			Controls (*n* = 28)		
	1st Postoperative CTA	Pre-Endoleak CTA	*p*-Value	1st Postoperative CTA	Matched CTA	*p*-Value
SAL, mm	11.2 (5.6–20.6)	3.9 (0.0–11.4)	**0.006**	21.3 (14.1–25.8)	25.4 (19.0–36.2)	**0.015**
SAL/neck length ratio	0.6 (0.3–1.1)	0.1 (0.0–0.4)	**0.001**	0.8 (0.6–1.8)	1.0 (0.8–1.9)	0.076
Shortest fabric distance, mm	1.2 (−1.3–7.4)	5.7 (2.0–12.3)	**<0.001**	1.0 (0.4–3.5)	5.0 (1.1–9.2)	**0.011**
Expansion, %	85.2 (82.1–90.4)	96.2 (92.7–101.3)	**<0.001**	88.6 (85.1–94.9)	89.9 (85.9–96.8)	0.23
Maximum aneurysm diameter, mm	64.3 (60.6–73.7)	65.3 (57.1–75.6)	0.77	62.8 (59.2–66.0)	52.0 (44.0–60.3)	**<0.001**

Data are presented as median (quartile 1–quartile 3). Boldface *p* values are statistically significant (*p* < 0.05). SAL: shortest apposition length.

**Table 3:** Differences in post-endovascular aneurysm repair endograft and aneurysm dimensions between the first postoperative computed tomography angiography (CTA) and the pre-endoleak/matched CTA.

	Type 1a Endoleak (*n* = 28)	Controls (*n* = 28)	*p*-Value
SAL, mm	−4.0 (−9.6 to 0.0)	4.0 (−1.6 to 10.8)	**<0.001**
SAL/neck length ratio	−0.2 (−0.6 to 0.0)	0.2 (−0.1 to 4.1)	**<0.001**
Shortest fabric distance, mm	3.7 (0.9 to 6.6)	1.2 (−0.9 to 5.3)	0.11
Expansion, %	8.8 (3.2 to 13.1)	2.4 (−4.1 to 6.4)	**<0.001**
Maximum aneurysm diameter, mm	0.6 (−4.3 to 5.0)	−8.5 (−17.4 to −4.1)	**<0.001**

Data are presented as median (quartile 1–quartile 3). Boldface *p* values are statistically significant (*p* < 0.05). SAL: shortest apposition length.

## Data Availability

The data presented in this study are available on request from the corresponding author. The data are not publicly available due to privacy or ethical restrictions.

## Supplementary Materials

The following supporting information can be downloaded at: https://www.mdpi.com/article/10.3390/jcm12123969/s1, Figure S1: Flowchart of patient selection for the current study in relation to the previous study by this research group.

## References

[1] Karathanos C., Spanos K., Saleptsis V., Ioannou C., Tsetis D., Kakissis J., Papazoglou K., Giannoukas A.D. (2016). One Year Outcome Using Newer Generation Endografts: A National Multicenter Study on Real Word Practice. Ann. Vasc. Surg..

[2] Salemans P.B., Lind R.C., van der Linde R.A., Pierie M.P., Fritschy W.M. (2021). Up to 10-year follow-up after EVAR with the Endurant stent graft system: A single-center experience. J. Cardiovasc. Surg..

[3] O’Donnell T.F., McElroy I.E., Mohebali J., Boitano L.T., Lamuraglia G.M., Kwolek C.J., Conrad M.F. (2022). Late Type 1A Endoleaks: Associated Factors, Prognosis and Management Strategies. Ann. Vasc. Surg..

[4] Antoniou G.A., Georgiadis G.S., Antoniou S., Neequaye S., Brennan J.A., Torella F., Vallabhaneni S.R. (2015). Late Rupture of Abdominal Aortic Aneurysm After Previous Endovascular Repair: A Systematic Review and Meta-analysis. J. Endovasc. Ther..

[5] Chaikof E.L., Dalman R.L., Eskandari M.K., Jackson B.M., Lee W.A., Mansour M.A., Mastracci T.M., Mell M., Murad M.H., Nguyen L.L. (2018). The Society for Vascular Surgery practice guidelines on the care of patients with an abdominal aortic aneurysm. J. Vasc. Surg..

[6] Wanhainen A., Verzini F., Van Herzeele I., Allaire E., Bown M., Cohnert T., Dick F., van Herwaarden J., Karkos C., Koelemay M. (2019). Editor’s Choice—European Society for Vascular Surgery (ESVS) 2019 Clinical Practice Guidelines on the Management of Abdominal Aorto-iliac Artery Aneurysms. Eur. J. Vasc. Endovasc. Surg..

[7] Andersson M., Sandström C., Stackelberg O., Lundqvist R., Nordanstig J., Jonsson M., Roy J., Andersson M., Hultgren R., Roos H. (2022). Structured CT analysis can identify the majority of patients at risk of post-EVAR rupture. Eur. J. Vasc. Endovasc. Surg..

[8] De Vries J.-P.P.M., Zuidema R., Bicknell C.D., Fisher R., Gargiulo M., Louis N., Oikonomou K., Pratesi G., Reijnen M.M.P.J., Valdivia A.R. (2023). European Expert Opinion on Infrarenal Sealing Zone Definition and Management in Endovascular Aortic Repair Patients: A Delphi Consensus. J. Endovasc. Ther..

[9] Schuurmann R.C.L., Overeem S.P., Van Noort K., De Vries B.A., Slump C.H., De Vries J.-P.P.M. (2018). Validation of a New Methodology to Determine 3-Dimensional Endograft Apposition, Position, and Expansion in the Aortic Neck After Endovascular Aneurysm Repair. J. Endovasc. Ther..

[10] Schuurmann R.C.L., Van Noort K., Overeem S.P., Van Veen R., Ouriel K., Jordan J.W.D., Muhs B.E., Mannetje Y.W., Reijnen M.M.P.J., Fioole B. (2018). Determination of Endograft Apposition, Position, and Expansion in the Aortic Neck Predicts Type Ia Endoleak and Migration After Endovascular Aneurysm Repair. J. Endovasc. Ther..

[11] Geraedts A.C.M., Mulay S., Vahl A.C., Verhagen H.J.M., Wisselink W., de Mik S.M., van Dieren S., Koelemay M.J., Balm R., Elshof J. (2022). Editor’s Choice—Post-operative Surveillance and Long Term Outcome after Endovascular Aortic Aneurysm Repair in Patients with an Initial Post-operative Computed Tomography Angiogram Without Abnormalities: The Multicentre Retrospective ODYSSEUS Study. Eur. J. Vasc. Endovasc. Surg..

[12] Von Elm E., Altman D.G., Egger M., Pocock S.J., Gøtzsche P.C., Vandenbroucke J.P., STROBE Initiative (2014). The Strengthening the Reporting of Observational Studies in Epidemiology (STROBE) Statement: Guidelines for reporting observational studies. Int. J. Surg..

[13] Geraedts A.C.M., Zuidema R., Schuurmann R.C.L., Kwant A.N., Mulay S., Balm R., de Vries J.-P.P., ODYSSEUS-T1EL Study Group (2022). Shortest Apposition Length at the First Postoperative Computed Tomography Angiography Identifies Patients at Risk for Developing a Late Type Ia Endoleak After Endovascular Aneurysm Repair. J. Endovasc. Ther..

[14] van der Riet C., de Rooy P.M., Tielliu I.F., Kropman R.H., Wille J., Narlawar R., Elzefzaf N.Y., Antoniou G.A., De Vries J.-P., Schuurmann R.C. (2021). Endograft apposition and infrarenal neck enlargement after endovascular aortic aneurysm repair. J. Cardiovasc. Surg..

[15] Baderkhan H., Haller O., Wanhainen A., Björck M., Mani K. (2018). Follow-up after endovascular aortic aneurysm repair can be stratified based on first postoperative imaging. Br. J. Surg..

[16] Gonçalves F.B., van de Luijtgaarden K.M., Hoeks S.E., Hendriks J.M., Raa S.T., Rouwet E.V., Stolker R.J., Verhagen H.J. (2013). Adequate seal and no endoleak on the first postoperative computed tomography angiography as criteria for no additional imaging up to 5 years after endovascular aneurysm repair. J. Vasc. Surg..

[17] Spanos K., Rohlffs F., Panuccio G., Eleshra A., Tsilimparis N., Kölbel T. (2019). Outcomes of endovascular treatment of endoleak type Ia after EVAR: A systematic review of the literature. J. Cardiovasc. Surg..

[18] Albertini J.-N., Kalliafas S., Travis S., Yusuf S.W., Macierewicz J.A., Whitaker S., Elmarasy N., Hopkinson B. (2000). Anatomical risk factors for proximal perigraft endoleak and graft migration following endovascular repair of abdominal aortic aneurysms. Eur. J. Vasc. Endovasc. Surg..

[19] Deery S.E., Ergul E.A., Schermerhorn M.L., Siracuse J.J., Schanzer A., Goodney P.P., Cambria R.P., Patel V.I. (2018). Aneurysm sac expansion is independently associated with late mortality in patients treated with endovascular aneurysm repair. J. Vasc. Surg..

[20] Candell L., Tucker L.-Y., Goodney P., Walker J., Okuhn S., Hill B., Chang R. (2014). Early and delayed rupture after endovascular abdominal aortic aneurysm repair in a 10-year multicenter registry. J. Vasc. Surg..

[21] van Prehn J., Schlösser F.J.V., Muhs B.E., Verhagen H.J.M., Moll F.L., van Herwaarden J.A. (2009). Oversizing of aortic stent grafts for abdominal aneurysm repair: A systematic review of the benefits and risks. Eur. J. Vasc. Endovasc. Surg..

[22] Gilling-Smith G.L., Martin J., Sudhindran S., Gould D.A., McWilliams R.G., Bakran A., Brennan J., Harris P. (2000). Freedom from endoleak after endovascular aneurysm repair does not equal treatment success. Eur. J. Vasc. Endovasc. Surg..

[23] Oliveira-Pinto J., Ferreira R.S., Oliveira N.F., Gonçalves F.B., Hoeks S., Van Rijn M.J., Ten Raa S., Mansilha A., Verhagen H. (2020). Morphologic chances and clinical consequences of wide aaa necks treated with 34–36 mm proximal diameter evar devices. Angiol. Cir. Vasc..

[24] Jordan W.D.J., Mehta M., Varnagy D., Moore W.M.J., Arko F.R., Joye J., Ouriel K., de Vries J.-P., Eckstein H., van Herwaarden J. (2014). Results of the ANCHOR prospective, multicenter registry of EndoAnchors for type Ia endoleaks and endograft migration in patients with challenging anatomy. J. Vasc. Surg..

[25] Kouvelos G.N., Oikonomou K., Antoniou G.A., Verhoeven E.L.G., Katsargyris A. (2017). A Systematic Review of Proximal Neck Dilatation After Endovascular Repair for Abdominal Aortic Aneurysm. J. Endovasc. Ther..

[26] Chatzelas D.A., Loutradis C.N., Pitoulias A.G., Kalogirou T.E., Pitoulias G.A. (2023). A systematic review and meta-analysis of proximal aortic neck dilatation after endovascular abdominal aortic aneurysm repair. J. Vasc. Surg..

[27] Oliveira N.F.G., Oliveira-Pinto J., van Rijn M.J., Baart S., Raa S.T., Hoeks S.E., Gonçalves F.B., Verhagen H.J. (2021). Risk Factors, Dynamics, and Clinical Consequences of Aortic Neck Dilatation after Standard Endovascular Aneurysm Repair. Eur. J. Vasc. Endovasc. Surg..

[28] Morris L., Stefanov F., Hynes N., Diethrich E.B., Sultan S. (2016). An Experimental Evaluation of Device/Arterial Wall Compliance Mismatch for Four Stent-Graft Devices and a Multi-layer Flow Modulator Device for the Treatment of Abdominal Aortic Aneurysms. Eur. J. Vasc. Endovasc. Surg..

[29] Malach L., Tehrani N., Kolachina S., Krawczyk K., Wozniak A., Soult M., Aulivola B., Bechara C.F. (2022). Effect of Stent-Graft Active Fixation and Oversizing on Aortic Neck Dilation After Endovascular Aneurysm Exclusion for Infrarenal Aortic Aneurysm. Ann. Vasc. Surg..

[30] Mirza T.A., Karthikesalingam A., Jackson D., Walsh S.R., Holt P.J., Hayes P.D., Boyle J. (2010). Duplex ultrasound and contrast-enhanced ultrasound versus computed tomography for the detection of endoleak after EVAR: Systematic review and bivariate meta-analysis. Eur. J. Vasc. Endovasc. Surg..

