# Supplementary material for: Diminishing Endograft Apposition during Follow-Up Is an Important Indicator of Late Type 1a Endoleak after Endovascular Aneurysm Repair
Source: J Clin Med. 2023 Jun 10;12(12):3969. doi: 10.3390/jcm12123969 (PMC10299238; doi:10.3390/jcm12123969)
Supplement: Supplementary file 1 [file jcm-12-03969-s001.zip › jcm-2393683-supplementary.pdf]

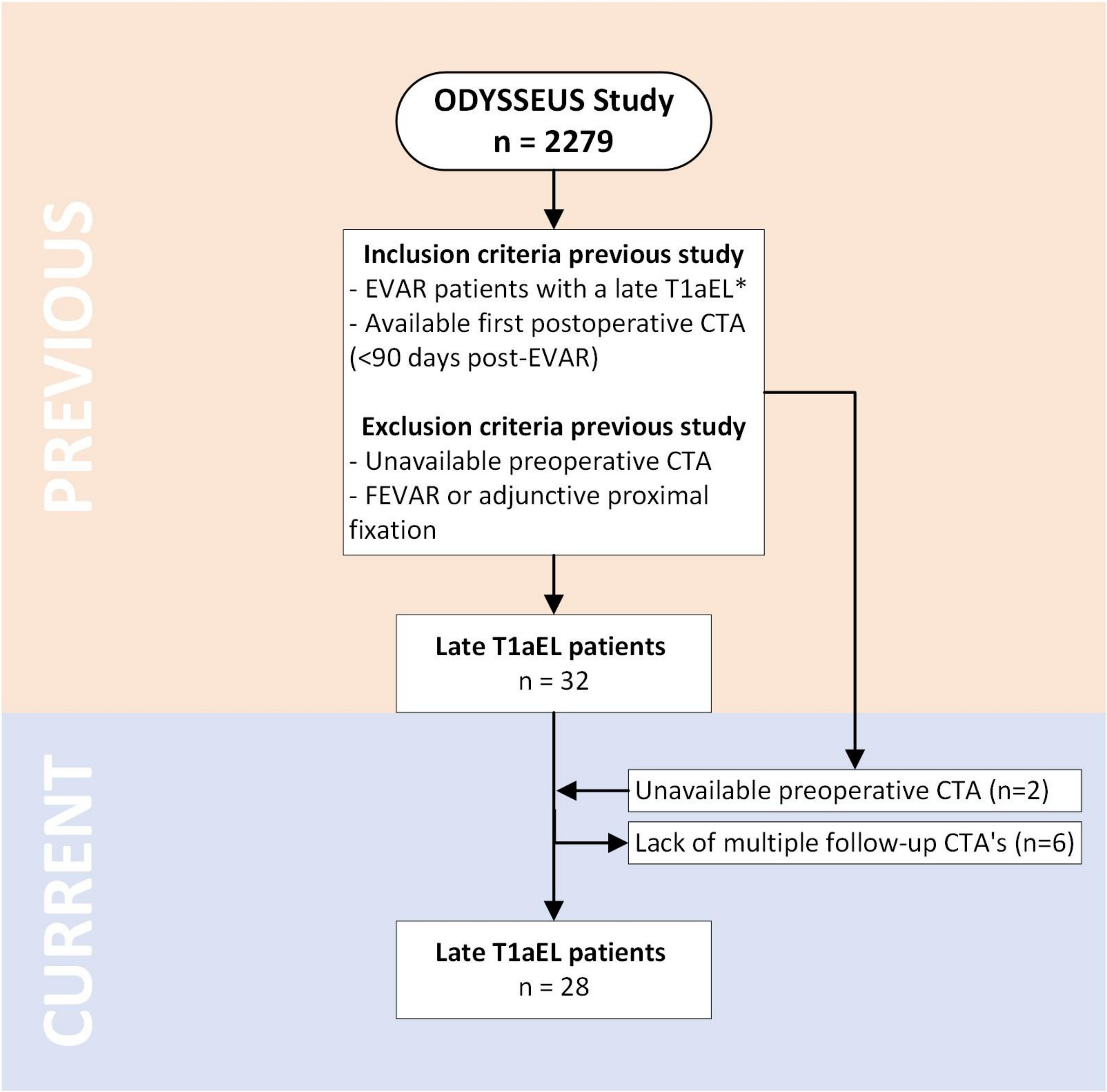


**Supplementary Figure S1.** Flowchart of patient selection for the current study in relation to the previous study by this research group. *CTA:* computed tomography angiography, *EVAR*: endovascular aneurysm repair, *FEVAR:* fenestrated endovascular aneurysm repair, *ODYSSEUS*: Observing a Decade of Yearly Standardised Surveillance in EVAR patients with Ultrasound or CT Scan, *T1aEL:* type 1a endoleak. *T1aEL in patients without abnormalities on the first postoperative CTA.
